# Fungal Biodegradation of Polyurethanes

**DOI:** 10.3390/jof9070760

**Published:** 2023-07-19

**Authors:** Clotilde Maestri, Lionel Plancher, Alexis Duthoit, Ronan L. Hébert, Patrick Di Martino

**Affiliations:** 1 Laboratoire ERRMECe, Cergy Paris University, 1 Rue Descartes, 95000 Neuville-sur-Oise, France; 2 Laboratoire GEC, Cergy Paris University, 1 Rue Descartes, 95000 Neuville-sur-Oise, France; ronan.hebert@cyu.fr; 3 SPPM—27 Rue Raffet, 75016 Paris, France; alexis.duthoit@sppm.fr

**Keywords:** polyurethane, polyester urethane, polyether urethane, deterioration, degradation, biodegradation, fungi, mold

## Abstract

Polyurethanes (PURs) are versatile polymers used in a wide variety of fields, such as the medical, automotive, textile, thermal insulation, and coating industries as well as many everyday objects. Many PURs have applications that require a long service life, sometimes with exposure to aggressive conditions. They can undergo different types of physicochemical and biological degradation, but they are not compostable, and many of them constitute persistent waste in the environment. Although both bacteria and fungi can be involved in the degradation of PURs, fungi are often the main biodegradation agents. The chemical structure of PURs determines their degree of biodegradation. Fungal biodegradation of PURs is linked to the production of enzymes, mainly esterases and proteases, alongside laccases, peroxidases, and tyrosinases, which can modify the structure of polyurethane compounds by forming carbonyl groups. The experimental analysis of the biodegradation of PUR can be carried out by bringing the polymer into contact with a mold in pure culture or with a microbial consortium. Then, global measurements can be taken, such as weight loss, tensile tests, or the ability of microorganisms to grow in the presence of PUR as the sole carbon source. The analysis of the chemical structure of the polymer and its degradation products after fungal growth can confirm biodegradation and specify the mechanism. The main avenues of future research are directed towards the development of fully biodegradable PURs and, on the contrary, towards the development of PURs that are more resistant to degradation phenomena, in particular biodegradation, for applications where the material is in contact with living organisms.

## 1. Introduction

Since their development in the 1930s, polyurethanes (PURs) have played an important role in a large variety of applications [1]. They are used in medical, automotive, textile, thermal insulative, coating, and everyday objects. PURs can be found in car seats, furniture, mattresses, clothing, waterproof coatings, paints, and pipes. This presence in many fields of application is linked to the versatility of polymers with urethane functions. However, polyurethanes usually have other functions (urea, ether, ester, aromatic, hydroxide, amine, etc.) and, thus, present a wide variety of physical or chemical properties.

Generally, urethane bonds -O-(C=O)-NH- are obtained by reactions between alcohols -OH and isocyanates -N=C=O. Polyurethane is a polymer containing several urethane bonds in its chain. To form a PUR, polyols, and diisocyanates are used. PURs are composed of a succession of hard and soft segments, themselves composed, respectively, of isocyanates and polyols. The nature of the polyol and the isocyanate determines the final properties of the polymer, such as its softness, hardness, and flexibility. Thus, the flexibility of PUR is increased by lengthening the chain of the polyol. Isocyanates are molecules with short chains that constitute areas of increased crystallization, hence the name “hard segments”. Isocyanate can be aliphatic, aromatic, or cycloaliphatic. The most commonly used diisocyanates are isophorone diisocyanate, toluene diisocyanate, and hexamethylene diisocyanate. Polyurethanes can be synthesized without isocyanate. Synthesis can be achieved in an aqueous solvent via transurethanization and aminolysis of cyclic carbonate. It consists of a unique reaction of cyclic carbonates with amines to form hydroxyurethanes. Polyols can be polyether for foams, polyester for thermoplastics, or polycarbonate for implanted biomaterials. We thus speak of polyether urethane (PEUR), polyester urethane (PESTUR), polycarbonate urethane (PCU), and non-isocyanate polyurethane (NIPU). There is another recent category of PURs, the green polyurethanes (GPU), that are obtained by reactions between more sustainable components. Waterborne polyurethanes (WBPU) differ from solvent-borne PURs in that the polymerization reaction takes place in an aqueous solution. WBPUs have a low odor and do not contain residual-free isocyanate. The simplest polyurethanes are linear, but there are branched PURs and crosslinked PURs, which are obtained by varying the choice of polyol and isocyanate [2].

Many PURs have applications that require a long service life, sometimes with exposure to aggressive conditions. They are thus subject to aging and even physicochemical and biological degradation phenomena. Sunlight, water, heat, organic and inorganic chemicals, and reactive oxygen species are all sources of degradation of PURs [3,4]. Like other synthetic polymers, PURs are significant environmental contaminants despite their usefulness for various human activities. It is essential to understand the mechanisms of their degradation in the environment or during waste treatment processes in order to ensure their functionality over time and limit their negative impact [5,6,7]. The mechanism and ease with which a PUR is biodegraded depend on its molecular composition and structure. The susceptibility of a polymer to biodegradation depends on its physical and chemical characteristics. The higher its molar mass and density, the less susceptible the polymer is to biodegradation. High hydrophobicity may limit the ability of some microorganisms to bind to the material, thus limiting their ability to degrade it. By limiting the accessibility of chains, crystallinity limits the biodegradability of a polymer [8]. A highly crosslinked polymer is also less prone to biodegradation. Some chemical bonds that make up a polymer may be more easily degradable than others and be a prime target for biodegradation initiation [9,10,11,12]. Hardness also influences the biodegradability of a polymer [13,14]. The biodegradation of a polymer takes place in several stages as follows: adhesion of cells or spores, growth of the biomass and biofilm development [15,16], fragmentation of the polymer, depolymerization by the action of enzymes and extracellular free radicals, intracellular metabolization, and finally mineralization. High-molecular-weight polymers cannot be transported across the cell membrane of microorganisms. Their degradation into low-molecular-weight polymers allows them to cross the cell membrane and be metabolized in microbial cells [11,17].

PURs are not biodegradable or compostable polymers according to NF EN 13432 and OECD (Organisation for Economic Co-operation and Development) 301B standards [18,19]. Thus, for a material to be considered biodegradable, it must be able to reach 90% biodegradation in less than 6 months, and after composting for 3 months, the total residue greater than 2 mm must be less than 10% of the initial mass. Alternatively, 60% of the material must be released as CO_2_ within 28 days of exposition to an inoculum from activated sludge, unchlorinated sewage effluents, surface waters, and/or soils. Nevertheless, some degree of biodegradation of PURs can be observed either with microbial consortia present in soil or with microorganisms in vitro in the laboratory.

This review deals with fungi involved in the biodegradation of PURs and the impact of this biodegradation on the functionality of materials and waste treatment.

## 2. Biodiversity of Fungi Involved in Polyurethane Degradation

Bacteria and fungi can be involved in the degradation of PURs, but fungi are often the main biodegradation agents. Pieces of PESTUR buried in the soil are mainly biodegraded by fungi such as *Geomyces pannorum*, *Nectria gliocladioides*, and *Penicillium ochrochloron* [20]. Although both are used to inoculate a waste treatment bioreactor, *Fusarium solani* is much more effective than *Pseudomonas* sp. in biodegrading polyurethane foam in vitro [6].

Various fungi such as *Alternaria*, *Aspergillus flavus*, *Aspergillus fumigatus*, *Aspergillus tubingensis*, *Chaetobium globosum*, members of the *Cladosporium cladosporioides* complex, *Curvularia senegalensis*, *Penicillium chrysogenum*, *Papiliotrema laurentii*, and *Pestalotiopsis* are capable of biodegrading PESTURS [10,21,22,23,24,25,26,27,28,29]. In addition to their activity towards polyester urethanes, *Alternaria*, *Aspergillus fumigatus*, *Aspergillus niger*, various species of *Cladosporium*, and *Penicillium chrysogenum* can also biodegrade PEURs [25,28,30,31].

Fungal biodegradation of PURs is linked to the production of enzymes inducible by the presence of the substrate. Esterase and protease are the two main families of enzymes involved in the biodegradation process, both of which are capable of hydrolyzing the urethane bond. Esterase can also degrade PESTUR by hydrolyzing the ester bond. Amidases are involved in PUR biodegradation by hydrolysis of amide bonds. Ureases can biodegrade PUR poly(ether urea) by mainly attacking urea bonds. Owen et al. described a urethane hydrolase produced by the soil fungus *Exophiala jeanselenei* involved in the biodegradation of urethane groups in Tolyl-carbamate urethane compounds [32]. Recent studies have explored the secretome of fungi capable of using PURs as their sole carbon source [33]. Proteases are the dominant enzyme group in *Fusarium* secretomes of different strains. The secretome of *Fusarium oxysporum* BPOP18 contains several hydrolases, such as acetylesterases, carboxypeptidases, cutinases, lipases, peptide hydrolases, and oxidoreductases. Oxidative enzymes can cleave C-C bonds. Laccases, peroxidases, and tyrosinases are thought to modify the structure of polyurethane compounds by forming carbonyl groups [33,34]. The fungal biodegradation process is increased when C=C double bonds are present in PUR [35].

The chemical structure of PURs determines their biodegradation under composting conditions [36]. As in other conditions of exposure to microbial attack, a PEUR is less susceptible to biodegradation than a PESTUR during composting. The increase in the number of hard segments or the presence of aromatic diisocyanates decreases the biodegradability of PUR, unlike that of aliphatic diisocyanates [5,36]. The biodegradation of PESTUR by thermophilic and thermotolerant fungi is observed at an increased rate during the thermophilic and early maturation phases of the composting process. Thus, the *Thermomyces lanuginosus* species, which produces numerous enzymes (thermostable proteases, amylases, xylanases, ureases, and lipases), are dominant on the surface of PESTUR coupons at 50 and 55 °C [5].

**Figure 1:**
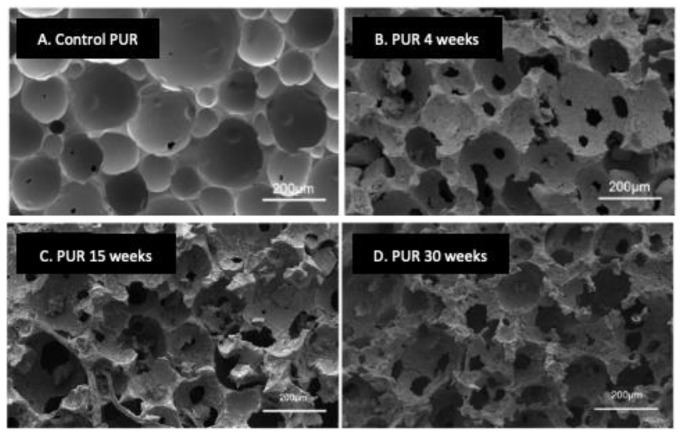
Scanning electron microscopy view of the biodegradation of PUR foam samples composed of linear polyester polyols based on aliphatic diacids and aliphatic diols incubated in a natural ocean environment over time. (**A**), the control sample was not immersed in the ocean. (**B**–**D**), samples were immersed in the ocean for 4, 15, or 30 weeks, respectively. Adapted from Ref. [39] which is an open access article under the CC BY license (http://creativecommons.org/licenses/by/4.0/).

Bio-based PESTUR and, in particular, vegetable oil-based PESTUR can be engineered to be fully biodegradable [37,38,39]. Such polymers can be rapidly biodegraded in compost, soil, and natural ocean environments by depolymerization, resulting in the release of original monomers that are then consumed during microbial growth. In the study by Gunawan et al., SEM imaging showed progressive degradation over time of biodegradable PUR samples during immersion in the ocean (Figure 1) [39]. This biodegradation resulted in increased porosity, crumbling, and cracking of the foam surface (Figure 1B–D) compared to the surface of a foam sample not submerged in the ocean (Figure 1A). Biodegradation of Starch-PUR films in soil occurs in successive steps as follows: wetting and colonization by microorganisms, hydrolysis of the starch part, fractionation of the polymer into small structures, and slow degradation of the polyurethane part [40].

Biodegradable PURs with anti-fouling properties have been described [41,42]. Marine biofouling is a major challenge for various human activities, including the performance and longevity of boat hulls. One of the most common approaches to combating marine biofouling is to develop anti-fouling coatings. Ma et al. have developed a biodegradable PUR whose soft segments are composed of dihydroxyl-terminated copolyester oligomers made of ε-caprolactone and glycolide [41]. The ester bond is the most sensitive zone to microbial attack, which allows the biodegradation of the material. The PUR material is designed to be eroded in the seawater environment, leading to a self-renewing and anti-fouling surface. Inorganic, organic, and living foulants attached to the coating detach from the surface during its degradation. The anti-biofouling activity of the degradable PUR depends on its rate of degradation; it increases with the rate of degradation for a glycolide content of 0 to 10% in moles. Anti-fouling efficiency and coating durability can be improved by adding the commonly used biocide 4,5-Dichloro-2-octylisothiazolone (DCOIT). Ali et al. have synthesised a biodegradable composite material corresponding to a polyurethane composed of ε-caprolactone, 4,4′-methylene bis(cyclohexyl isocyanate), and 1,4 butanediol, incorporating DCOIT and clay [42]. The adhesion strength and rate of degradation of the material are increased following the addition of clay by reducing the spherulite size and crystallinity of the polycaprolactone (PCL), which improves the amorphous interfacial region of the composite material. DCOIT molecules localized in the amorphous region give the material anti-adhesion properties against microorganisms. By reducing the size of the PCL, spherulite in the composite, the clay facilitates the enzymatic attack of the material.

Conventional PURs with anti-adhesion properties among microorganisms can be obtained by modifying the surface of the material [43,44]. Weintraub et al. coated the surface of a polyurethane catheter with a copolymer of astaxanthin (ATX) and polyethylene glycols (PEG) to develop an implantable medical device resistant to infections [43]. ATX is a xanthophyll carotenoid with antimicrobial properties. ATX can be polymerized with different dicarboxylic acid co-monomers. The biodegradation of the polymer coating in vivo and the antimicrobial properties of the material depend on the size of the PEG block. p(ATX-co-PEG 2000) is rapidly biodegraded and has no satisfactory antimicrobial properties in vivo. p(ATX-co-PEG 1000) and p(ATX-co-PEG 250) have antimicrobial properties in vivo that are negatively correlated with their rate of biodegradation. The slower the biodegradation rate, the more gradual the release of ATX and the better the antimicrobial properties. De La Franier et al. used another strategy to develop a PUR that reduces the adhesion of microorganisms to the surface of indwelling urinary catheters [44]. The anti-fouling material was a monolayer of monoethylene glycol hydroxide (MEG-OH) covalently bound to PUR via a siloxane network. After in vitro exposure for 24 to 72 h at 37 °C, a decrease in adherent bacteria varying from 85 to 96% was observed depending on the species tested, and a decrease of 90% was observed for *Candida albicans*. Moreover, only scattered microorganisms and small clusters of a few adherent cells formed on the MEG-OH coating without the development of biofilm, contrary to the strong formation of biofilm on the control PUR. A similar level of anti-adhesion was maintained after autoclaving or storing the material in the air for 4.5 months. The mechanism of the microbial anti-adhesion effect is unknown, but the anti-fouling activity of MEG-OH for blood and proteins is associated with the formation of an interfacial layer of hydration due to ether groups in the middle of chain [45].

## 3. Experimental Analysis of Polyurethane Biodegradation

**Figure 2:**
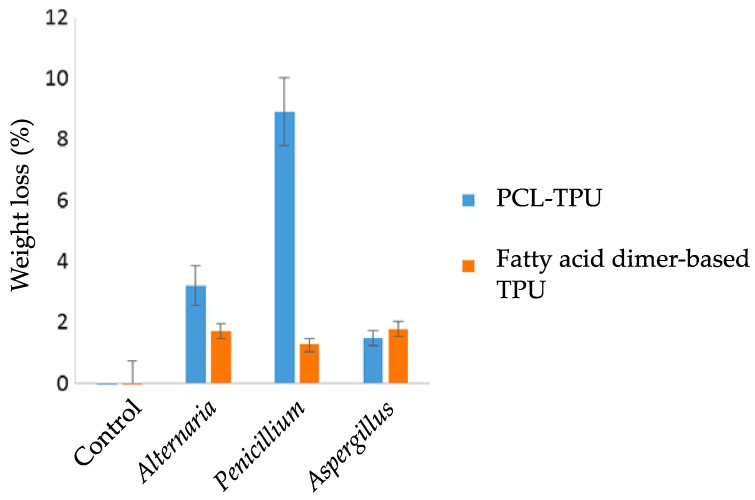
Weight loss measurements of thermoplastic polyurethane polycaprolactone (PLC-TPU) and TPU based on fatty acid dimers after 2 months of incubation at 30 °C with different molds. Control: material incubated in the absence of molds. Adapted from Ref. [46] which is an open access article under the terms of the Creative Commons Attribution License (http://creativecommons.org/licenses/by/4.0/).

The weight loss measurement is a global analysis, which constitutes the first global approach for studying the biodegradation of PURs [46,47]. This technique is not highly sensitive and requires significant biodegradation of the polymer to be efficient. In the study by Magnin et al., thermoplastic polyurethanes had a mass loss of a few percent (9% maximum) after exposure for two months at 30 °C to strains of *Alternaria*, *Penicillium,* or *Aspergillus* (Figure 2) [46]. In order not to underestimate biodegradation, it is essential to rid the material of all the biomass that has developed on its surface. For this, washes with mechanical action and chemical treatments with ethanol, a non-ionic surfactant, or sodium hypochlorite can be used [27,28,47].

To identify microorganisms that biodegrade PURs, colloidal model polymers like Impranil-DLN^®^ can be used. Impranil-DLN^®^ is an ester-urethane that is easily degradable and assimilated by microorganisms. It can induce the fungal secretion of PUR-degrading enzymes active against both PESTUR and PEUR. Impranil-DLN^®^ forms a whitish, opaque surface when dispersed in an agar-supercooled medium [23,26,48]. Its biodegradation leads to the appearance of translucent zones around the microbial colonies. A biodegradation test can also be carried out in a liquid medium. The turbidity measurement correlated to the concentration of impranil-DLN^®^ makes it possible to assess its degradation after 14 days of incubation in the presence of mold [28]. These tests based on the disappearance of colloidal-polymer-related opaque cloudiness can reveal the degradation of a PUR by enzymatic hydrolysis of urethane bonds but also of ester bonds [49].

In the study by Darby and Kaplan (1968), about 100 PURs of different chemical compositions were subjected to a mixture of molds containing several species of *Aspergillus*, *Penicillium*, *Trichoderma*, and *Chaetomium globosum* (Table 1) [21]. The polyurethanes were synthesized from various dialcohols or polyesters such as 1,5-Pentanediol, 3-Methyl-2,4-pentanediol, 2-Methyl-2,4-pentanediol, Diethylene glycol, Dipropylene glycol, Polypropylene glycol, Polyethylene glycol adipate, Poly-1,3-propanediol adipate, and various diisocyanates, including tolylene-2,4-diisocyanate, diphenylmethane-4,4′-diisocyanate, 3,3′-bitolylene-4,4′-diisocyanate, and 1,6-hexamethylene-diisocyanate. These polymers were then placed on agar plates for individual testing. The mold mixture was inoculated, and growth was observed after 2–3 weeks at 30 °C. The growth rate was assessed visually. The mold growth rate defines the ability to use the polymer as a nutrient source. A high growth rate, therefore, corresponds to the high biodegradation of the polymer. PESTURs were shown to be more easily degraded by molds than PEURs, and biodegradation occurred more favorably in the presence of a sufficiently long carbon chain between the urethane bonds. The greater sensitivity of PESTURs to biodegradation has been confirmed by other studies [9,10,22,23,24,25,26,27,28].

**Figure 3:**
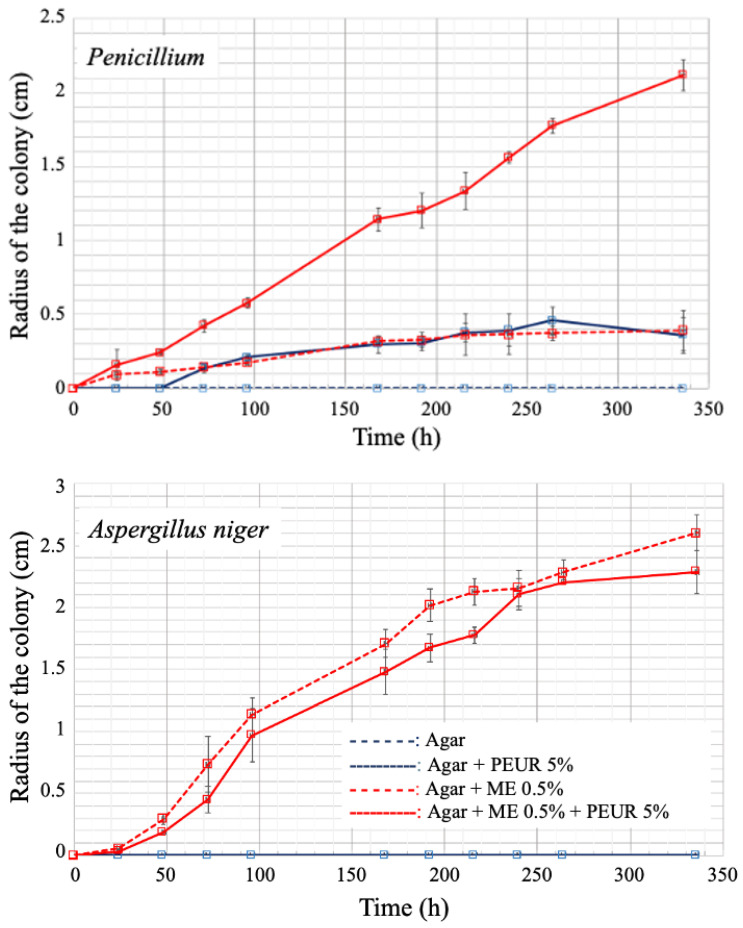
Comparative growth of molds in Petri dishes containing different agar media over time at 24 °C. ME, malt extract. PEUR, polyether-urethane polymer [31].

In the study by Plancher et al., a translucent ground PEUR was incorporated into supercooled agar or supercooled agar containing low-concentration malt extract [31]. Comparative growth of molds on these different media showed the ability of a *Penicillium* sp. strain to metabolize PEUR as a sole nutrient source or in the presence of other nutrients at low concentrations (Figure 3). Thus, the diameter of *Penicillium* colonies exceeded 2 cm in diameter after 350 h at 24 °C in the presence of malt extract and PEUR, whereas it was less than 0.5 cm in the presence of malt extract or PEUR alone. In the same conditions, *Aspergillus niger* only grew in the presence of malt extract; its growth rate was not modified by the presence of PEUR.

After its biodegradation, an analysis of the chemical composition of the polymer and the appearance of its degradation products can be performed. After 14 days of growth of *Cladosporium pseudocladosporioides* strain T1.PL.1 in a minimal medium containing Impranil-DLN^®^, FTIR analysis of cell-free filtrates revealed a decrease in signals corresponding to the carbonyl group (C=O) and the urethane bond (C-N-H) [28]. The analysis of the compounds present in the incubation medium was carried out by GC-MS in the presence and absence of mold. In the absence of mold, minor changes in some compounds were observed. On the other hand, significant changes in the profile of the compounds were observed after fungal growth, with some disappearing and others appearing. This confirmed a decrease in compounds with the ester bond and an increase in alcohols and hexane diisocyanate, which highlighted the biodegradation of Impranil-DLN^®^. Detection of aromatic amines released during fungal biodegradation of Tolyl-carbamate urethane compounds can also be performed by GC-MS to confirm the cleavage of urethane groups [32]. In addition to revealing the biodegradation of a PUR, FTIR analysis can also help identify the mechanism. Significant decreases in band intensities at 890 (C-C stretch), 960 (C-C stretch), 1080 (C-O stretch in C-O-C=O of urethane), 1240 (asymmetric C-O-C stretch, CN stretch, CH_2_ twist), 1275 (asymmetric C-O-C stretch, CN stretch, CH_2_ torsion), 1330 (CH_2_ wagging), and 1465 cm^−1^ (CH_2_ wagging), and a significant increase in the band at 1710 cm^−1^ (C=O urethane H-bounded) were observed by Plancher et al. after 11 days of growth of *Penicillium* on an agar medium containing the PEUR PUX1520 [31]. The same experiment carried out with a strain of *Aspergillus niger* showed few changes in the IR spectrum of PEUR except for an increase in the signal at 1710 cm^−1^. Each of these two strains proved capable of biodegrading PEUR PUX1520 after 4 weeks of incubation at 24 °C in a broth containing the polymer and malt extract. A closer analysis of the carbonyl group and full-spectrum IR signals revealed oxidative degradation of the polymer [31]. It has long been known that photooxidation is a major factor in the degradation of polyether-based polyurethanes [50]. Oxidation is also a major factor in the microbial biodegradation of polyether urethanes [31,51,52,53].

**Figure 4:**
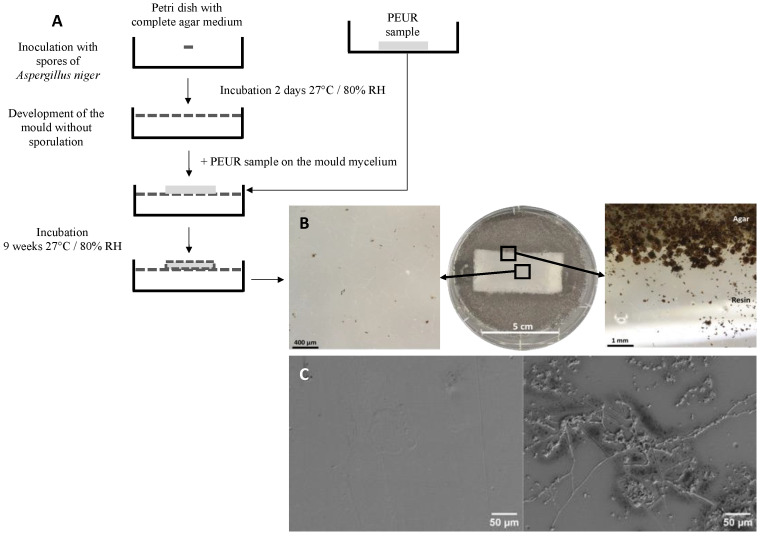
(**A**), Diagram of method B′ of the ISO 846 standard. In this method, the PUR sample is placed on the agar nutrient medium containing a carbon source when it is completely covered by mycelium. The test was performed in a 90 mm diameter Petri dish. (**B**), Growth of *Aspergillus niger* on polyether urethane after 9 weeks of contact according to the method described in A The magnification was obtained with a stereomicroscope. (**C**), scanning electron microscope views of a PUR coupon incubated on an agar medium containing no carbon source (**left** picture) and of a coupon of the same material incubated under the same conditions in the presence of *A. niger* (**right** picture).

The ISO 846 standard for the determination of the deterioration of plastics when exposed to microorganisms is well suited to the study of the biodegradation of PURs [18]. In this standard, indirect or direct deterioration caused by microorganisms is determined by visual observation, measurement of mass changes, and measurement of changes in other physical properties like surface gloss, bending properties, impact resistance, and hardness. It is only applicable to materials with a flat surface. It includes five main methods for determining the resistance of plastics to fungi (method A), fungistatic effects (methods B and B′), resistance to bacteria (method C), and resistance to soil microorganisms (method D). Figure 4 shows a fungal growth test on a PEUR according to ISO 846, methods B′ and A. Strong growth of *Aspergillus niger* was observed on the polymer in different conditions. The higher spore density at the edges of the polymer than elsewhere on the agar medium was observed macroscopically (Figure 4B). The presence of mycelial filaments running along the surface of the polymer and spores reaching the center of the polymer were observed under light and scanning electron microscopy (Figure 4B,C).

**Figure 5:**
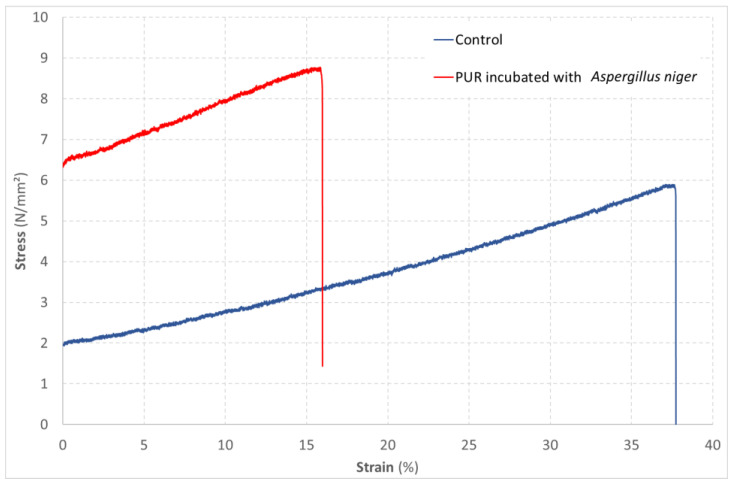
Evolution of the strain of a PUR material as a function of the stress applied in a tensile test. Comparison of an unexposed material (control) with a material exposed to the growth of *Aspergillus niger* according to method B′ of the ISO 846 standard.

Among the physical property changes associated with biodegradation, the mechanical strength of materials in tensile tests can be measured [54,55]. Exposure of polyurethane elastomer films to a strain of *Chaetomium globosum* for 90 days at 28 °C induces a decrease in breaking stress and tensile strength [52]. Figure 5 shows the comparison of the behavior of a PUR material with or without exposure to the growth of *A. niger*, according to ISO 846, method B′, in a tensile test according to the ISO 527-2 standard [56]. After exposure to *A. niger*, an increase in the maximum stress of 6.9 to 8.8 N/mm^2^ and a decrease in strain of 37 to 16% before failure were observed (Figure 5).

**Figure 6:**
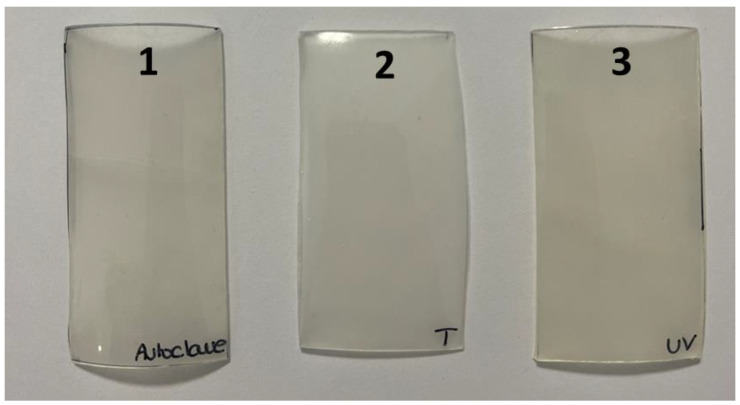
Macroscopic observation of PUR coupons (6 × 3 cm) after different physical treatments. 1, autoclaving 15 min at 121 °C. 2, no treatment (control). 3, UV-C exposure for 24 h at 254 nm.

One of the difficulties of protocols for studying PUR biodegradation during fungal growth in vitro is the sterilization of the material before exposure. Although it does not induce mass loss and can be used for foams, autoclaving can induce alterations in many PURs [28,57]. UV irradiation can be used, but this can also degrade the chemical structure and mechanical properties of PUR [4,58]. Thus, UV irradiation can induce breaks in the polymer chain at the molecular level and yellowing, cracks, and holes at the macroscopic scale. Figure 6 shows the macroscopically visible yellowing of PUR after autoclaving or UV-C irradiation. Colorimetric analysis of the coupons indicated E values of 84.61, 83.53, and 80.53 for the control, autoclaved, and UV-C irradiated samples, respectively. The color difference ΔE*ab between the control and the UV-C-treated PUR was 4.9.

**Figure 7:**
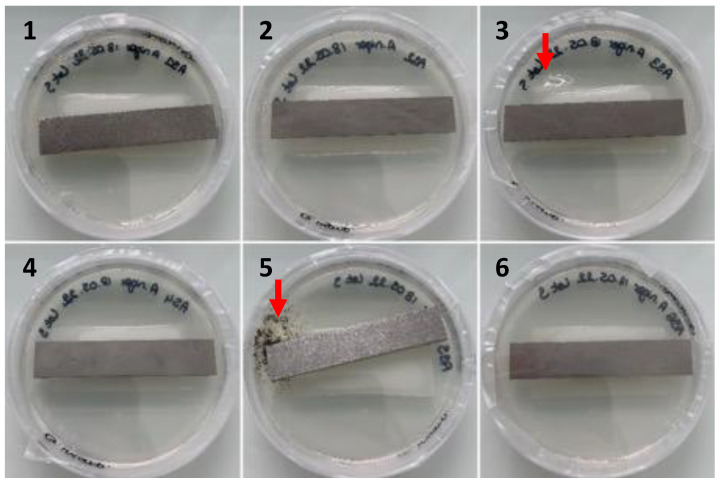
Observation of the growth of contaminants (red arrows, Petri dishes 3 and 5) developing from PUR coupons deposited on nutrient agar (Petri dish 9 cm in diameter) after cleaning with ethanol and disinfection with o-phenylphenol after 6 weeks of incubation at 27 °C/80% relative humidity according to ISO 846.

An ethanol rinse has been used in some studies to sterilize PUR coupons before exposure [24,27]. The Standard ISO 846 recommends cleaning polyurethane coupons in a 30% ethanol bath and, in some cases, disinfecting coupons in an o-phenylphenol bath at a concentration of 1 g/L [18]. A solution of o-phenylphenol 0.1% (weight/volume) and 79% ethanol (volume/volume) in water was effective against a mixture of *Staphylococcus aureus*, *Mycobacterium bovis*, spores of *Trichophyton mentagrophytes*, and Sabin poliovirus type 1 but not against spores of *Bacillus stearothermophilus* [59]. By using the cleaning/disinfection protocol recommended by standard ISO 846, the persistence of bacterial and fungal contaminants on PUR coupons is frequently observed (Figure 7). In the example shown in Figure 7, there was no growth of contaminants from PUR coupon numbers 1, 2, 4, and 6, while bacterial growth and fungal growth were observed from coupon numbers 3 and 5, respectively. However, the six coupons underwent the same cleaning and disinfection protocol.

**Figure 8:**
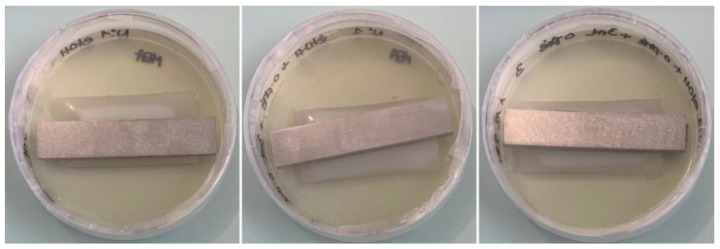
Observation of cleaned and disinfected PUR coupons placed on malt extract agar (Petri dish 9 cm in diameter), incubated for 6 weeks at 27 °C/80% relative humidity. Before depositing on agar plates, the coupons were cleaned with distilled water and disinfected with successive baths of 70% ethanol and 1% o-phenylphenol.

Adaptations of the protocols recommended by the ISO 846 standard make it possible to improve the decontamination efficiency of the surface of PUR coupons. Figure 8 shows the result obtained after the application of a disinfection protocol composed of a sterile distilled water bath, followed by an ethanol bath at its bactericidal concentration of 70% [60], and a bath of o-phenylphenol at an increased concentration of 1% (weight/volume) effective against microorganisms in vegetative and spore form [61]. Incubation on agar was carried out in the presence of a 1% o-phenylphenol solution on the PUR coupons. After the application of this protocol to PUR coupons and 6 weeks of incubation, the absence of contaminants was demonstrated (Figure 8). This is an important point of the ISO 846 standard, which requires testing a batch of incubated material without inoculation by microorganisms.

## 4. Conclusions

Molds are major agents in the biodegradation of PURs. They express this activity within complex communities in soils or composts, and many of them biodegrade polyurethanes in vitro in monoculture. The fungal biodegradation of PURs is mainly linked to the joint secretion of several enzymes that cleave the urethane bond or other bonds in the polymer either by hydrolysis or oxidation. Knowledge of the mechanisms of this biodegradation can be used to develop materials resistant to fungal biodegradation that are intended for long-term exposure to microorganisms, like waterproofing coatings, or, on the contrary, to develop biodegradable PURs that are compostable or have anti-fouling properties. The development of PURs with anti-fouling properties is another key issue because microbial adhesion and the development of biofilm on polymers are essential steps in their biodegradation or the development of infections. Research must also focus on the isolation, characterization, and optimization of microbial strains or microbial consortia with high potential for the biodegradation of PURs and the optimization of composting processes.

## Figures and Tables

**Table 1:** Growth of a mixture of molds observed on polyurethanes.

Diol	Diisocyanate				
	Tolylene-2,4-Diisocyanate	Diphenylmethane-4,4′-Diisocyanate	3,3′-Bitolylene-4,4′-Diisocyanate	1,6-Hexamethylene-Diisocyanate	
1,5-Pentanediol	3	3	2	1	Polyurethane
3-Methyl-2,4-pentanediol	2	1	1	1	Polyurethane
2-Methyl-2,4-pentanediol	2	1	1	1	Polyurethane
Diethylene glycol	1	1	1	0	Polyurethane
Dipropylene glycol	0	0	0	0	Polyurethane
Polypropylene glycol 400	2	2	2	2	Polyether urethane
Polypropylene glycol 1020	3	3	3	3	Polyether urethane
Polypropylene glycol 1320	3	2	3	2	Polyether urethane
Polyethylene glycol adipate	4	4	4	4	Polyester urethane
Poly-1,3-propanediol adipate	4	4	4	4	Polyester urethane

(Growth rates were assessed visually by scoring: 0 = no growth, 1 = trace of growth visible under the microscope, 2 = slight growth, 3 = moderate growth, 4 = significant growth. Based on Darby and Kaplan 1968).

## Data Availability

Data sharing not applicable.
